# Mandibular advancement devices decrease systolic pressure during the day and night in patients with obstructive sleep apnea: A systematic review and meta-analysis

**DOI:** 10.1007/s11325-023-02984-0

**Published:** 2024-01-05

**Authors:** Alba Belanche Monterde, Álvaro Zubizarreta-Macho, Ana Belén Lobo Galindo, Alberto Albaladejo Martínez, José María Montiel-Company

**Affiliations:** 1 Department of Surgery, Faculty of Medicine and Dentistry, University of Salamanca, 37008 Salamanca, Spain; 2 Department of Implant Surgery, Faculty of Health Sciences, Alfonso X El Sabio University, 28691 Madrid, Spain; 3 Department of Stomatology, Faculty of Medicine and Dentistry, University of Valencia, 46010 Valencia, Spain

**Keywords:** Blood pressure, MAD, Mandibular advancement device, Hypertension, Obstructive sleep apnea syndrome, OSA

## Abstract

**Abstract:**

The aim of this systematic review and meta-analysis was to analyze whether or not mandibular advancement devices (MADs) produce changes in blood pressure in patients with obstructive sleep apnea (OSA) in relation to use time and if the device is used at night or day.

**Materials and method:**

A systematic review of the literature and meta-analysis was carried out in accordance with PRISMA guidelines. In the bibliographic search, a total of four databases were consulted: PubMed-Medline, Scopus, Web of Science, and Cochrane. Of the 622 articles initially revealed, 160 duplicates were eliminated. After applying the selection criteria, 17 articles were included for the qualitative analysis and 4 for the meta-analysis. The studies were combined using a random effects model with the inverse method of variance, determining the mean differences in systolic and diastolic pressure before and after treatment using the MAD splint as the effect size. Day/night circadian effect and treatment time were analyzed using meta-regression with a mixed-effects model.

**Results:**

MAD treatment was not found to affect diastolic pressure. By combining the four studies with the control group in a meta-analysis (*I*^2^ = 75%; *z* =  − 0.15; *p*-value = 0.882), the mean difference in diastolic pressure between the MAD group and the control group was estimated at − 0.06 (− 0.86; 0.74). The meta-regression also showed no significant effect of day/night (*p* = 0.560) or treatment time (*p* = 0.854) on diastolic pressure. When combining the four studies with the control group (*I*^2^ = 84%%; *z* =  − 1.47; *p*-value = 0.142), a non-significant mean difference in systolic pressure between the MAD group and the control group of − 0.99 (− 2.31; 0.33) was estimated in the meta-analysis. However, when assessing the effect of day/night or treatment time on systolic blood pressure using a meta-regression, the latter showed significant covariates that reduce systolic blood pressure values in the model at night (*p* < 0.001) and in relation to treatment time (*p* < 0.001).

**Conclusions:**

Only systolic pressure appears to be affected by the use of the MAD in patients with OSA, and this decrease in systolic pressure is greater at night and when treatment time is longer.

## Introduction

Obstructive sleep apnea syndrome (OSA) is a condition in which respiratory ventilation is reduced or completely halted during sleep due to obstruction or collapse of the upper airway [1]. Hypopnea refers to an oxygen desaturation of at least 3% [2]. The severity of this disorder is categorized using the apnea–hypopnea index (AHI) [3]. The etiology of OSA is multifactorial, with anatomical, functional, and genetic factors all having interacting effects. The upper airway collapses due to an imbalance between relevant forces, inducing the collapse [4, 5]. The genioglossus muscle, which comprises most of the tongue, is one of the dilator muscles of the upper airway and it is important that its patency is maintained during sleep. In patients with OSA, reduction of the motor endplate and tissue changes in the genioglossus muscle are observed [6, 7]. The most widely used treatments for OSA are continuous positive airway pressure (CPAP), intraoral devices, and dietary measures for weight loss. One of the most commonly used intraoral devices is the mandibular advancement device (MAD) [7–9]. MADs increase the volume of upper airway by positioning the mandible and tongue in an anterior position. Different MAD designs have been used for the treatment of OSA, which can be grouped into mono-block and bi-block devices. MADs can also be custom-made or pre-manufactured and titratable or not titratable. Bi-block devices are commonly titratable and consist of two separated parts for each dental arch that are joined together in different ways. They can be connected in the lateral or the frontal area of the jaws [10]. The effects of MADs on skeletal and dental position have also been studied. Some studies have found a reduction in overbite and overjet and proclination of mandibular incisors with the use of MADs. Reductions in interincisal angle and retroclination of maxillary incisors have also been observed, but these results remain controversial. When studying the effectiveness of MADs, custom-made and titratable devices are more effective in patients with OSA, with greater adherence than found with CPAP machines [11]. Although CPAP has shown better results than MADs in preventing apnea–hypopnea events, MADs have provided similar improvements in quality of life and in cardiovascular variables when compared with CPAP [12]. OSA is a risk factor for hypertension and other associated morbidities such as angina, heart attack, or death. Hypertension is linked to the alteration of the sympathetic nervous system in these patients, which can trigger endothelial and metabolic dysfunction and inflammatory processes [1, 13]. Alteration of the sympathetic nervous system is a marker of risk for cardiovascular disease [14].

The aim of this systematic review and meta-analysis was to analyze whether or not MADs produce changes in blood pressure in patients with OSA in relation to use time.

## Materials and method

### Study design

This bibliographic search was conducted in accordance with PRISMA (Preferred Reporting Items for Systemic Reviews and Meta-Analyses; http://www.prisma-statement.org) guidelines for systematic reviews and meta-analyses (INPLASY registration number: INPLASY202270018). The review also met the criteria of the PRISMA 2009 Checklist [15].

### Focused question

The PICO (population, intervention, comparison, outcome) question was “Do MADs produce a reduction in systolic and diastolic blood pressure during day- and nighttime in patients with OSA?” with the following population: patients with OSA, adult patients, patients with hypertension, and patients treated for OSA using a MAD. Patients treated with a CPAP device were excluded.

### Databases and search strategy

An electronic search was conducted using the following electronic databases: PubMed, Scopus, Web of Sciences, and Cochrane (A.B.M. and A.Z.-M.). The search covered all internationally published literature from 1998 to 2022. The search included seven medical subject heading (MeSH) terms: “obstructive sleep apnea,” “blood pressure,” “hypertension,” “mandibular advancement device,” “apnea,” “MAD,” “OAS,” “heart,” and “sleep apnea.” The Boolean operators applied were (‘OR’ and ‘AND’). The search terms were structured as follows: (((oral AND devices) OR (oral AND appliances) OR (MAD) OR (mandibular AND advancement)) AND (sleep AND apnea) OR (OSA)) AND ((blood AND pressure) OR (heart) OR (hypertension) AND (randomized AND trial))). Two researchers (A.B.M. and A.Z.-M.) conducted a separate database search independently of each other. Titles and abstracts were selected by applying the inclusion and exclusion criteria.

### Study selection

Titles and abstracts were selected by two researchers (A.B.M. and J.M.M.-C.) by applying the inclusion and exclusion criteria.

Inclusion criteria are as follows: randomized clinical trials (RCT), with samples of patients aged from 25 to 65 years old, although most studies involved patients with a mean age of about 52 years; patients treated for OSA with MADs; and follow-up period of at least 1 month. No restriction was placed on year of publication or language.

Exclusion criteria were as follows: systematic literature reviews; clinical cases or case series; non-randomized clinical trials; pediatric patients; and patients treated with CPAP devices or another treatment. The following data were extracted from each article by two researchers (A.B.M. and J.M.M.-C.): author and year of publication; title and journal in which the article was published; sample size for the control group, sample size for the treatment group, pre- and postoperative blood pressure values (mmHg) in the control group and treatment group, expressed as mean and standard deviation (SD); follow-up time (months); and the variables studied.

### Data extraction and study outcomes

Data extraction was conducted separately by two different researchers (A.B.M. and J.M.M.-C.) using predefined Excel spreadsheets and accounting for the following items: author and year of publication; study type; sample size; follow-up time in months; pre- and postoperative blood pressure values; and sample size for the control and treatment groups.

### Methodological quality assessment

The risk of bias in the studies selected for review was assessed by two researchers (A.B.M. and A.Z.-M.) using the Jadad scale for methodological quality assessment of clinical trials. The Jadad scale consists of five items that evaluate randomization, researcher and patient blinding, and description of losses during follow-up, resulting in a score of 0–5. Scores of less than 3 are considered low quality [16]. The level of agreement between the evaluators’ scores was determined using Kappa scores.

### Quantitative synthesis—meta-analysis

Statistical data collection and analysis were performed by two researchers (A.Z.-M. and J.M.M.-C.). The studies included in the meta-analysis were combined using a random effects model with the inverse variance method. Effect size was calculated as the mean difference between the systolic and diastolic values of the study group compared with the control group at the end of the study period. The 95% confidence interval and prediction interval were calculated for all estimated variables.

Meta-regression was carried out using the mixed-effects method, with the moderator analysis used to assess the effect of day/night and treatment time.

Heterogeneity between the combined studies was assessed using the *Q*-test (*p*-value < 0.05) and quantified with the *I*^2^, considering slight heterogeneity to be between 25 and 50%, moderate heterogeneity to be between 50 and 75%, and high heterogeneity when greater than 75%. Statistical significance was assessed using the *Z* test (*p*-value < 0.05). Meta-analyses were represented using a forest plot and the meta-regression was represented with a bubble plot. Publication bias was assessed using the trim-and-fill adjustment method and represented using a funnel plot. Analysis of meta-evidence was conducted using the R software.

## Results

### Flow diagram

**Fig. 1:**
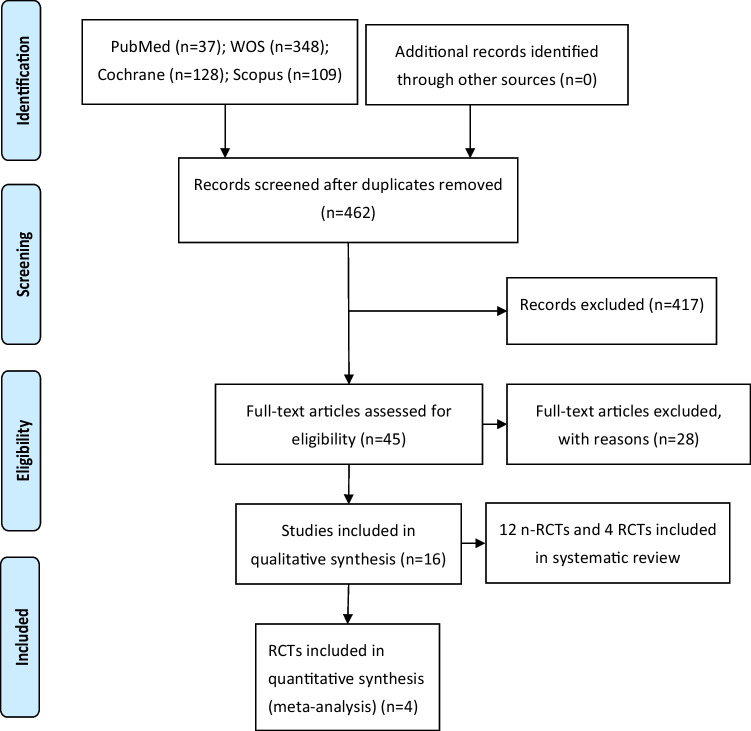
Preferred Reporting Items for Systematic Reviews and Meta-Analyses (PRISMA) flow diagram

The initial electronic search identified 37 articles in PubMed, 128 in Cochrane Library, 348 in Web of Science, 109 in Scopus, and none in gray literature. Of the total of 622 works, 160 were discarded as duplicates. After reading the titles and abstracts, a further 35 were eliminated, leaving a total of 45. A further 28 were rejected as they failed to fulfill the following inclusion criteria: minimum follow-up period; expression of blood pressure values in mmHg with mean and SD; and inclusion of a control group (intraoral device without mandibular advancement). A final total of 17 articles were included in the qualitative synthesis. Only four articles were included in the quantitative synthesis, as these included all the required data and variables. In the meta-analysis, only RCTs that showed results of treatment with MADs in patients with OSA were included. The authors selected only those RCTs that had a control group with an oral device without mandibular advancement or placebo device, a study group with MADs, and that showed separate results for daytime blood pressure and nighttime blood pressure. (Figure 1).

### Qualitative analysis

Of the 16 articles included [17–32], 11 were randomized clinical trials [14–18, 21, 23–26, 29] and 6 were non-randomized clinical trials [19, 20, 22, 27, 28]. The articles were all published between 2004 and 2021. Articles that compare pre- and postoperative blood pressure values with MADs but without a control group were included in the qualitative analysis. There were accepted ranges and mean values. The sample size ranged from 15 [20] to 169 [22]. Table 1 shows the results of this qualitative analysis.

**Table 1:** Qualitative analysis of articles included in the systematic review

Authors	Journal	Publication year	Study design	*n* total sample	*n* control group	*n* treatment group	Variables analyzed	Mean difference in pre- and postoperative SBP (mmHg) and cardiometabolic changes	Follow-up
Dissanayake et al. [14]	Sleep Med	2021	RCT	92	NAv	92	SBP, DBP, AHI, level of cooperation	ΔSBP day 0.20 [− 2.44; 3.84] ΔSBP night − 0.40 [− 4.42; 3.62]	1 month
Guimaraes et al. [15]	J Clin Sleep Med	2021	RCT	79	20	19	SBP at day- and nighttime, DBP at day- and nighttime	ΔSBP day − 0.80 [− 13.21; 11.61] ΔSBP night: − 0.90 [− 15.78; 13.98]	12 months
Hedberg P. et al. [16]	J Sleep Res	2021	RCT	72	36	36	SBP, DBP, BMI, cholesterol, AHI, triglycerides	AHI reduction. No changes in inflammatory biomarkers	3 months
Yamamoto et al. [17]	Heart Vessels	2019	RCT	40	NAv	40	SBP	ΔSBP day 1.30 [− 6.03; 8.63] ΔSBP night 1.60 [− 7.34; 10.54]	1 month
Rietz H. et al. [18]	J Am Heart Assoc	2018	RCT	96	43	41	SBP at day- and nighttime, DBP at day- and nighttime, AHI	ΔSBP day 1.30 [− 2.93; 5.53] ΔSBP night 1.40 [− 2.71; 5.51]	4 months
Van Haesendonck G. et al. [19]	Sleep	2017	NRCT	17	NAv	17	SBP, DBP, AHI	No changes in 24-h blood pressure	6 months
Galic T. et al. [20]	Sleep and Breath	2016	NRCT	15	NAv	15	SBP, DBP, cholesterol, insulin I, insulin II, HbA1c, glucose IE	AHI and cardiometabolic risk factors reduction. No significant changes in SBP	12 months
Glos et al. [21]	Sleep and Breath	2016	RCT	48	NAv	24	SBP, DBP	Beneficial changes in blood pressure during daytime	3 months
Sekizuka H. et al. [22]	Clin. Exp. Hypertens	2016	NRCT	169	NAv	68	SBP, DBP	ΔSBP − 2.4 [− 17.2; 12,4] ΔDBP − 2.0 [− 13.7; 9.7]	2 months
Dal-Fabro et al. [23]	Sleep Breath	2014	RCT	29	29	29	SBP at day- and nighttime, DBP at day- and nighttime, vitamins, level of cooperation	ΔSBP day − 2.90 [− 4.34; − 1.46] ΔSBP night − 3.50 [− 4.84; − 2.16]	3 months
Andren A. et al. [24]	Sleep Breath	2013	RCT	66	32	34	SBP at day- and nighttime, DBP at day- and nighttime	ΔSBP day 2.30 [− 2.55; 7.15] ΔSBP night 2.50 [− 3.92; 8.92]	3 months
Andren A. et al. [25]	J Oral Rehabil	2009	RCT	22	NAv	22	SBP, DBP	ΔSBP − 15.4 [− 34.1; 3.3] ΔDBP − 10.3 [− 20.3, 0.3]	3 years
Gauthier L. et al. [26]	Sleep Med	2009	RCT	16	NAv	16	SBP, DBP, sleep variables	Not statistical changes in SBP and DBP (*p* > 0.001)	3 months
Zhang L. et al. [27]	Zhonghua Yi Xue Za Zhi	2009	NRCT	46	21	25	SBP, DBP, AHI	Statistical decrease of SBP and DBP (*p* < 0.001)	3 months
Yoshida K [28]	Int J Prosthodont	2006	NRCT	161	NAv	161	SBP, SBP	Statistical decrease of SBP, DBP, and mean blood pressure (*p* < 0.001)	1 month
Gotsopoulos H. et al. [29]	Sleep	2004	RCT	64	NAv	64	SBP at day- and nighttime, DBP, sleep variables, AHI	ΔSBP day 4.40 [3.79; 5.01] ΔSBP night − 0.40 [− 0.97; 0.17]	2 months

*NRCT* non-randomized clinical trial, *RCT* randomized controlled trial, *NAv* not available, *SBP* systolic blood pressure, *DBP* diastolic blood pressure, *AHI* apnea–hypopnea index, *BMI* body mass index

### Quality assessment

The scores of the methodological quality assessment using the Jadad scale were calculated by one researcher (A.Z.-M.) and are shown in Table 2. The Jadad scale defined five articles as “Not applicable” because they did not involve randomized clinical trials [19, 20, 22, 27, 28]. Most of the randomized articles obtained 3 points, indicating medium methodological quality [14, 16, 17, 23, 24]; however, one article obtained 4 points, indicating medium–high quality [18]. Quality was most often scored lower due to the absence of double-blinding (Table 2).

**Table 2:** Assessment of methodological quality using the Jadad scale

Jadad criteria						
Author/year	Is the study described as randomized?	Is the study described as double-blinded?	Was there a description of withdrawals and dropouts?	Was the method of randomization adequate?	Was the method of blinding appropriate?	Score
Dissanayake et al. [14]	1	N/A	1	1	N/A	3
Guimaraes et al. [15]	1	N/A	1	N/A	N/A	2
Hedberg P [16]	1	N/A	1	N/A	N/A	3
Yamamoto et al. [17]	1	N/A	1	1	N/A	3
Rietz H. et al. [18]	1	1	N/A	1	1	4
Van Haesendonck G. et al. [19]	N/A	N/A	N/A	N/A	N/A	0
Galic T. et al. [20]	N/A	N/A	N/A	N/A	N/A	0
Glos et al. [21]	1	N/A	N/A	N/A	N/A	1
Sekizuka H. et al. [22]	N/A	N/A	N/A	N/A	N/A	0
Dal-Fabro et al. [23]	1	N/A	1	1	N/A	3
Andren A. et al. [24]	1	1	N/A	1	N/A	3
Andren A. et al. [25]	1	N/A	N/A	N/A	N/A	1
Gauthier L. et al. [26]	1	N/A	N/A	N/A	N/A	1
Zhang L. et al. [27]	N/A	N/A	N/A	N/A	N/A	0
Yoshida K [28]	N/A	N/A	N/A	N/A	N/A	0
Gotsopoulos H. et al. [29]	1	N/A	1	N/A	N/A	2

*N/A* not applicable

### Quantitative analysis

#### Systolic pressure

**Fig. 2:**
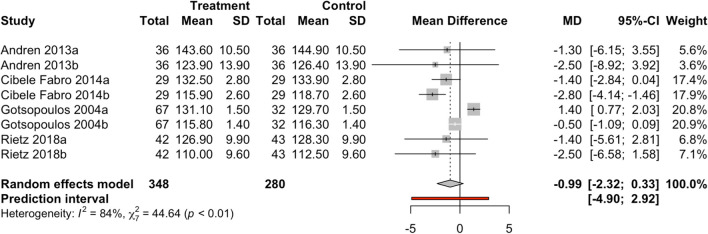
Forest plot of systolic pressure in the MAD vs. control groups. a = day; b = night

To assess the effect size on systolic blood pressure, eight results with day- and nighttime results from four studies were combined using a random-effects model (Fig. 2) with a total of 628 observations, estimating a statistically non-significant mean difference (*z* =  − 1.47; *p*-value = 0.143) between the two groups of -0.99 (CI-95% between − 2.32 and 0.33). The results were taken into account separately for the analysis of day- and nighttime. The meta-analysis found high levels of heterogeneity (*I*^2^ = 84.3%; *Q*-test = 44.6; *p*-value < 0.001).

**Fig. 3:**
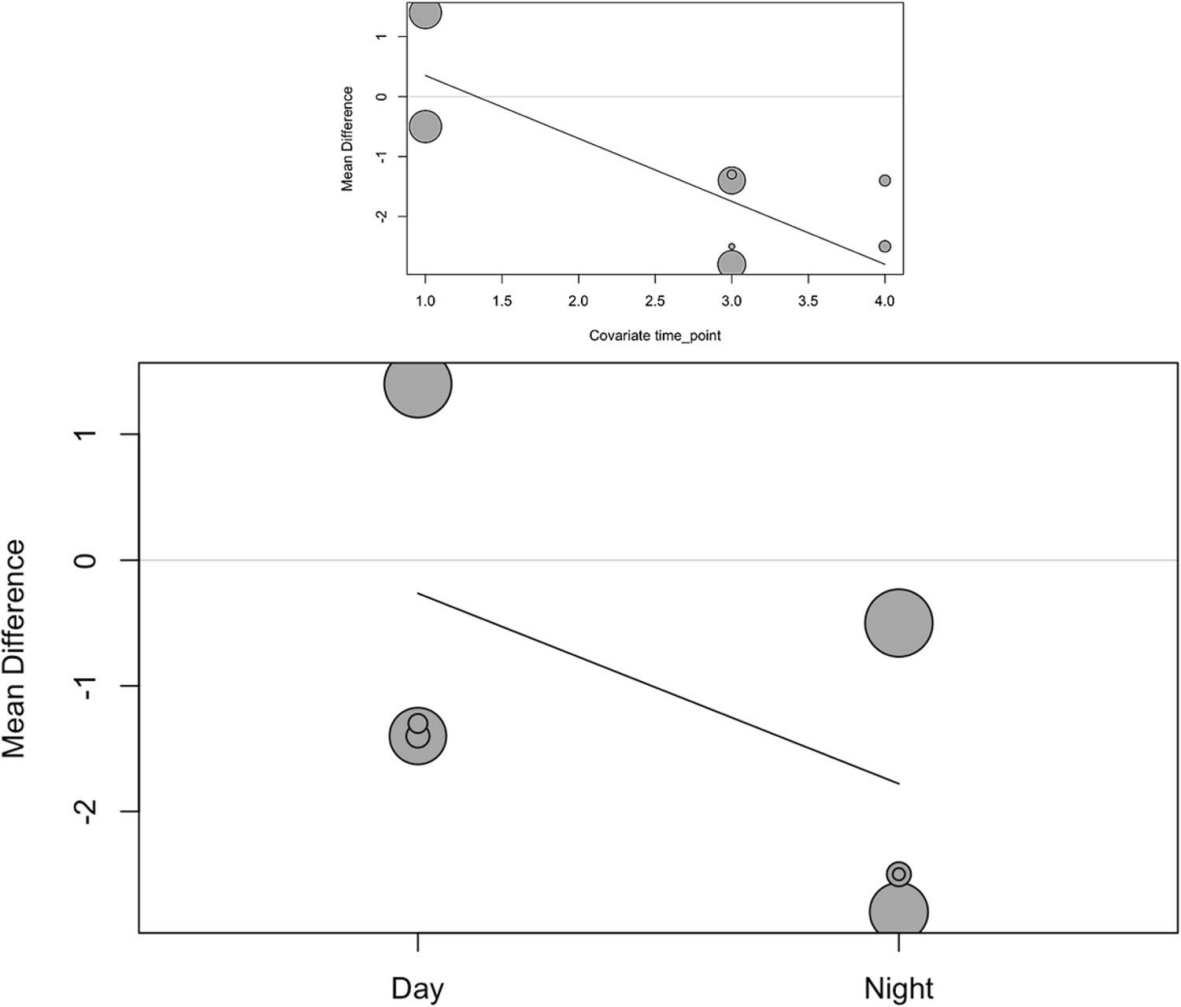
Bubble plots of systolic pressure meta-regression for time point and day/night

In order to analyze the effect size of day/night and treatment time (time point), the studies were combined in a meta-regression using a mixed-effects model. For systolic blood pressure, the estimation was an intercept of 2.50, as well as a slope for day/night of − 1.80 and slope for time point of − 1.17 (Fig. 3). The meta-regression did not show heterogeneity (*I*^2^ = 0%). The moderator analysis was significant with *p* < 0.001, indicating a significant effect on the reduction of systolic blood pressure for day/night (*p* < 0.001) and for time point (*p* < 0.001).

#### Diastolic pressure

**Fig. 4:**
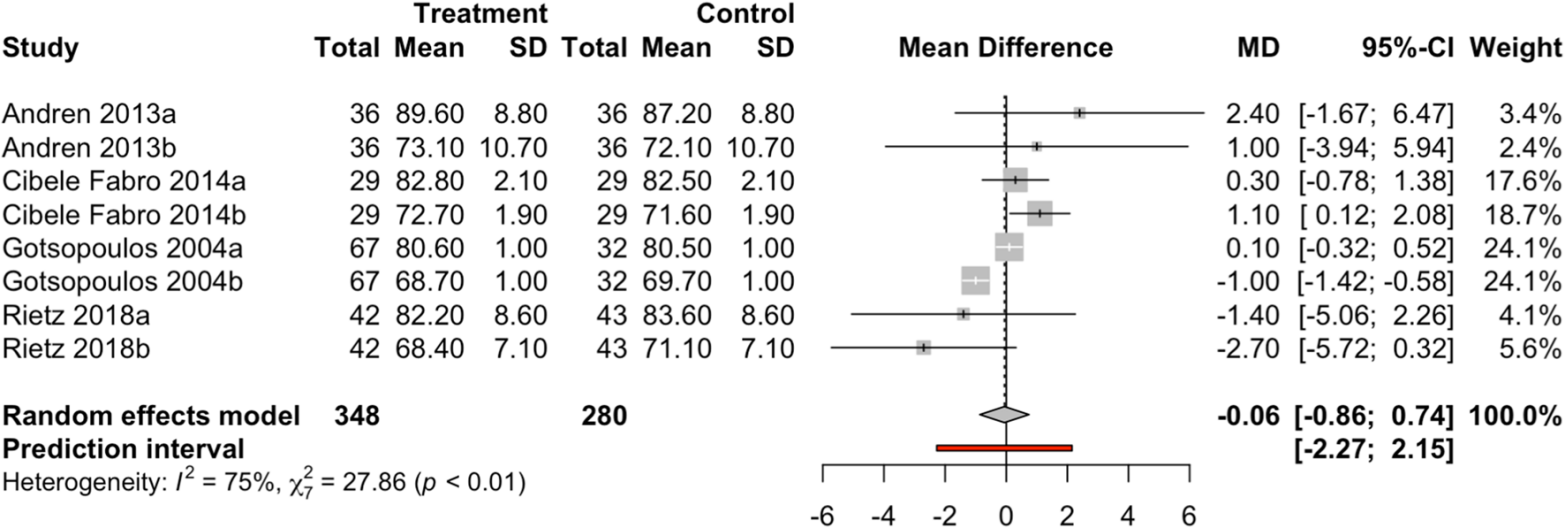
Forest plot of diastolic pressure in the MAD vs. control groups. a = day; b = night

To evaluate the effect size on diastolic blood pressure, eight results with day- and nighttime results from four studies were combined using a random-effects model (Fig. 4) with a total of 628 observations, estimating a statistically non-significant mean difference (*z* =  − 0.15; *p*-value = 0.883) between the two groups of − 0.06 (CI-95% between − 0.86 and 0.734). The meta-analysis showed high heterogeneity (*I*^2^ = 74.9%; *Q*-test = 27.8; *p*-value < 0.001).

**Fig. 5:**
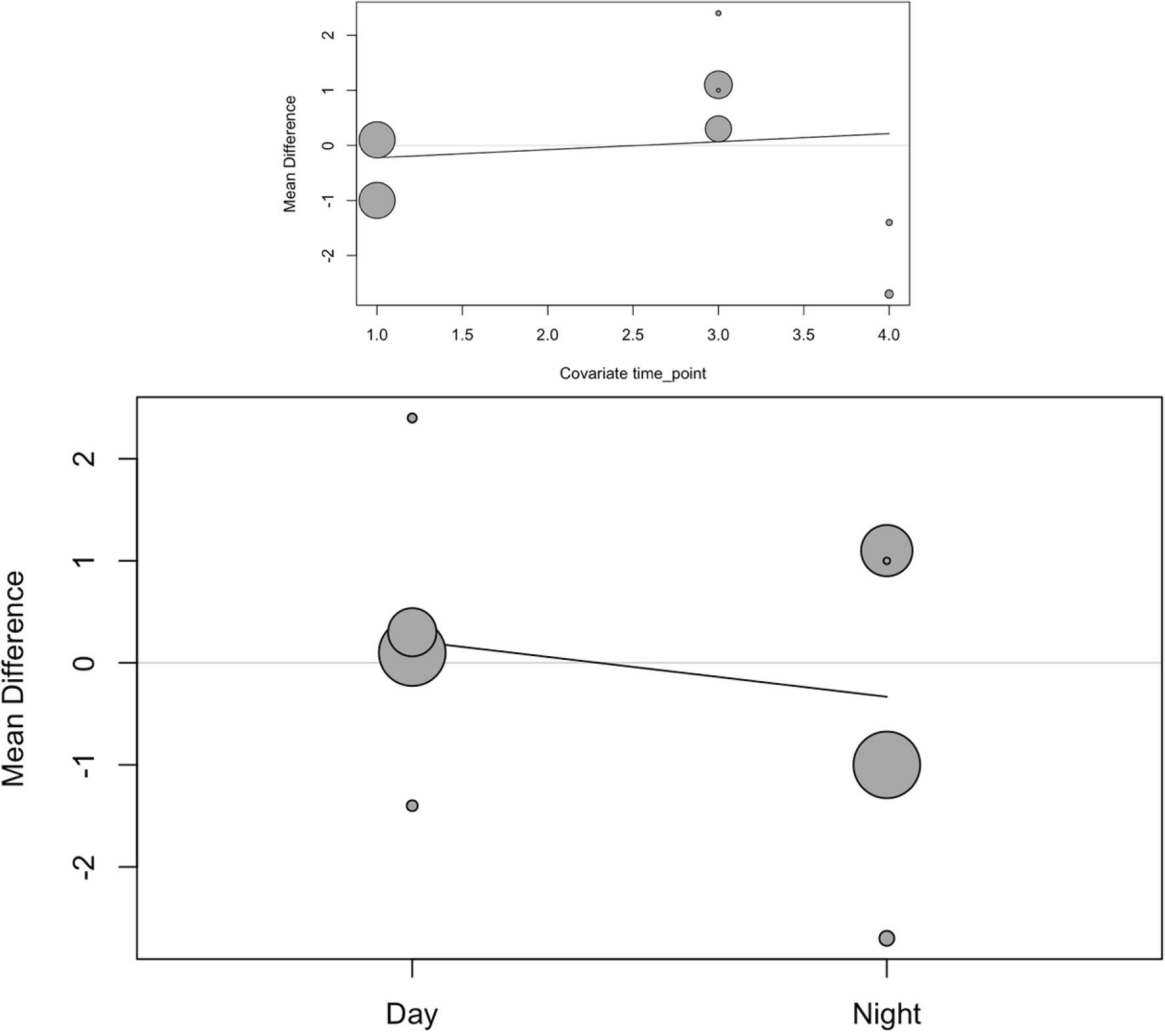
Bubble plots of diastolic pressure meta-regression for time point and day/night

For the diastolic blood pressure meta-regression, the same analysis was carried out. The estimation was an intercept of 0.05, as well as a slope for day/night of − 0.56 and slope for time point of 0.07 (Fig. 5). The meta-regression showed moderate heterogeneity (*I*^2^ = 62.8%). The moderator analysis found no statistical significance with *p* = 0.832, showing an absence of significance in the reduction of diastolic blood pressure for both day/night (*p* = 0.560) and for time point (*p* = 0.855).

### Publication bias

**Fig. 6:**
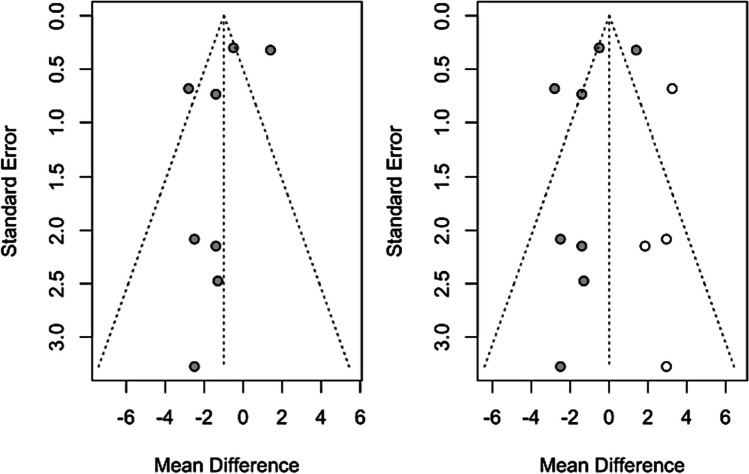
Trim-and-fill analysis of systolic pressure

**Fig. 7:**
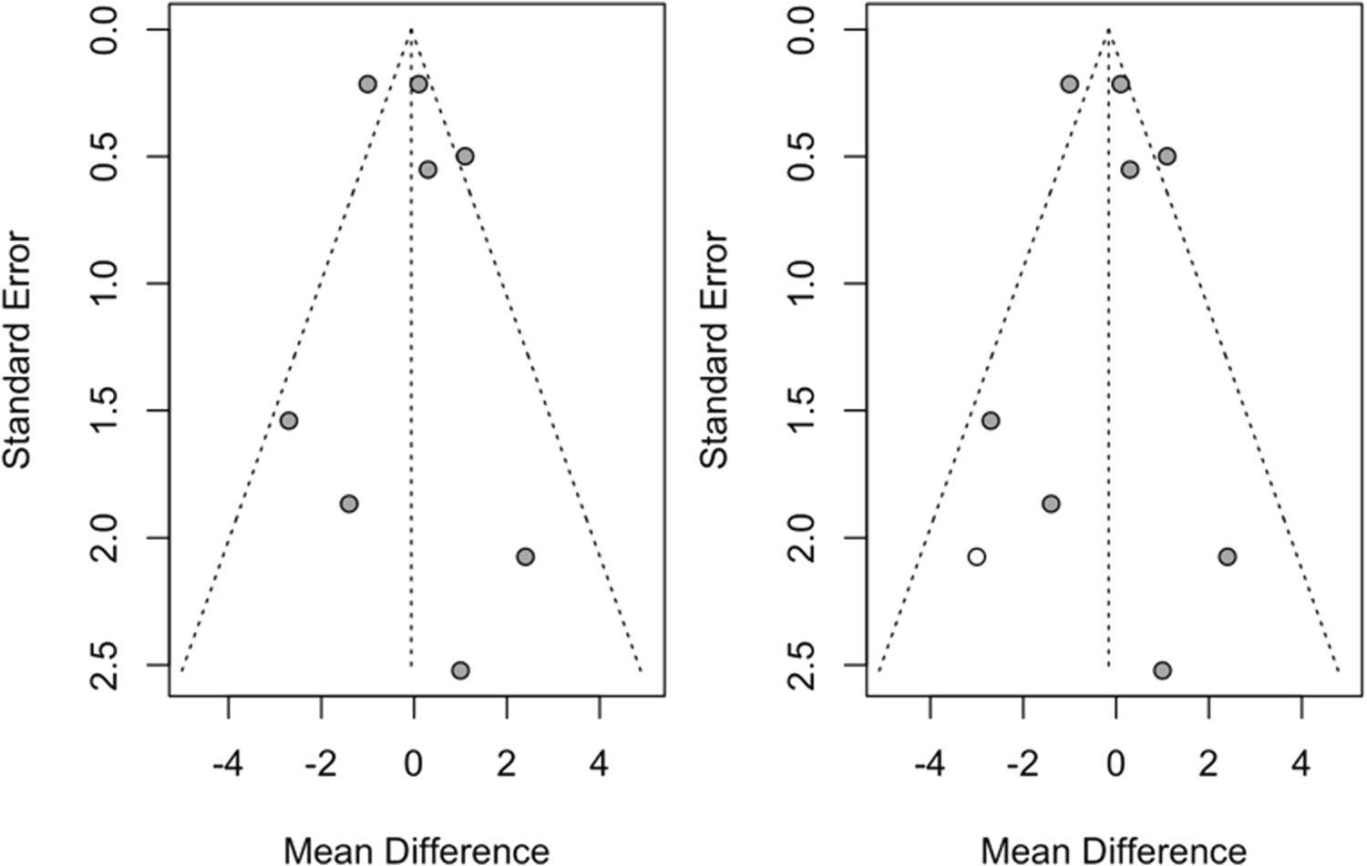
Trim-and-fill analysis of diastolic pressure

Figures 6 and 7 show the results of the trim-and-fill. To calculate the effect size on systolic blood pressure, four studies were added to adjust for funnel plot asymmetry. The mean difference estimate was calculated as 0.013 (CI-95% between − 1.364 and 1.391); *z* = 0.02; *p* = 0.985, finding no statistically significant difference from the initial − 0.99 (CI-95% between − 2.31 and 0.33), indicating little presence of publication bias for this meta-analysis (Fig. 6).

With regard to calculating the effect size on diastolic tension, only one study was added to adjust for funnel plot asymmetry. The mean difference estimate was calculated as − 0.159 (CI-95% between − 0.963 and 0.645); *z* =  *− *0.39; *p* = 0.697, finding no statistically significant difference from the initial − 0.06 (CI-95% between − 0.86 and 0.74), indicating little presence of publication bias for this meta-analysis (Fig. 7).

## Discussion

The results obtained from the current study accepted the null hypothesis (H_0_) that blood pressure is not altered with treatment.

The results obtained from the current study did not find any statistically significant differences when comparing the treatment group and the control group. Therefore, the variable was not reduced significantly with the use of MADs, although systolic blood pressure appeared to reduce with longer treatment time. The meta-regression found a lack of heterogeneity in systolic blood pressure results and significant effect of the day/night and treatment time variables on systolic blood pressure. However, these results were not obtained for diastolic blood pressure, indicating heterogeneity and no relationship with the variables evaluated.

### Risk factors for OSA

The incidence of OSA has been high for the last 40 years, having been observed in 25% of men and 11% of women [33]. Previous studies have identified both anatomical and functional factors linked to the development of OSA including smaller pharyngeal airways, bulky soft tissue, and lower-positioned hyoid bones. In addition, obesity has also been described as a relevant risk factor for the development of OSA. Leptin has also been highlighted as a functional risk factor because the presence of this macromolecule worsens OSA and has been demonstrated to affect sleep architecture, particularly in patients with hypertension [30, 34], as it affects satiety, glucose levels, and fatty acid metabolism. In addition, some evidence suggests that hypertension may be caused by OSA. It has been reported that blood pressure increases linearly as OSA worsens, regardless of gender or age [31]. Additionally, OSA is a risk factor for atherosclerosis and has been linked to atrial fibrillation in a dose–response relationship. OSA has also been associated with strokes and heart failure [35], as intermittent hypoxia can result in increased mean blood pressure and cerebral vascular pressure. Furthermore, OSA may lead to autonomic, hemodynamic, and biochemical changes [31, 32].

### OSA association with atherosclerosis

During sleep, parasympathetic nervous system activation is increased, but sympathetic nervous system is not. However, patients with OSA do exhibit increased activation of the sympathetic nervous system. Apnea leads to an increase in renal and adrenal hormones such as catecholamines, renin, and angiotensin II, affecting the renin-angiotensin system. Changes in systolic and diastolic blood pressure because of intermittent hypoxia have been observed [30, 31]. This effect activates the hypothalamic-pituitary adrenal axis, and the cortisol stress hormone is often elevated. Cortisol is also higher in hypertensive patients, but the relationship between OSA and hypertension is complex. In normotensive patients, treatment for OSA produces little reduction in blood pressure [31]. CPAP therapy has resulted in a 2–3 mmHg reduction in hypertensive patients. CPAP may therefore be beneficial in hypertensive patients with OSA. Even so, it has not been confirmed that CPAP can prevent cardiovascular events. CPAP brings with it the issue of poor adherence. About 30% of patients never initiate treatment, and 25% abandon treatment within the first year of starting [31]. Atherosclerosis is more common in older patients due to the decreased thickness and diminished elasticity of big vessels [36]. Patients with OSA tend to be older, as the lower muscle tone found in the elderly favors collapse of the upper airway [30, 33]. Hypertension in the elderly is caused by atherosclerosis, and only systolic blood pressure is increased. This may explain why this meta-analysis found that only systolic blood pressure seems to be reduced when using a MAD. In addition, a meta-analysis of studies in young patients published by Ai et al. observed that systolic blood pressure was elevated in moderate and mild cases of OSA. The author suggested that OSA is a risk factor for adverse systolic blood pressure and its prevention is therefore important [37]. In the included studies, the age of the subjects depended on each study evaluated, with patients averaging around 52 years old [18, 19, 27], whereas other studies included patients ranging from 25 to 65 years old [24], a wide age range when taking into account the degenerative cardiovascular processes that occur in these patients over time [34]. A previous meta-analysis found that oral appliance therapy could modestly decrease systolic and diastolic blood pressure, but without reaching statistical significance (*p* > 0.001). *I*^2^ values were elevated, showing heterogeneity of the articles included [34]. However, in these results, the initial articles observed decreases of − 2 mmHg in diastolic blood pressure and a reduction of 17% in the prevalence of hypertension [38, 39]. Another meta-analysis in which the effects of CPAP on blood pressure was studied found a − 4.11 mmHg reduction in systolic blood pressure during the day and a − 3.17 mmHg reduction at night. The reduction of diastolic blood pressure was − 2.11 mmHg. However, these changes were also not statistically significant [40]. Surgical treatments such as adenotonsillectomy have been studied in children. This treatment is a first-line therapy in pediatric patients. When adenotonsillectomy effects were studied, a reduction of − 6.23 mmHg in mean systolic blood pressure and − 7.93 mmHg in mean diastolic blood pressure was observed. The pediatric patients with hypertension as a concomitant illness obtained better results than normotensive patients [41]. It has also been reported that treatment with CPAP produce a decrease in cortisol levels of − 0.39 and a reduction in systolic blood pressure of − 5.4 mmHg [42]. In the present study, a reduction of − 0.99 (*p* = 0.143) in mean systolic blood pressure and − 0.06 (*p* = 0.734) in mean diastolic blood pressure were obtained in adult patients.

### MAD as a treatment for OSA

Many treatment options have been indicated in patients with OSA. The first-line treatment has been CPAP, but due to the issues with adherence, OSA is also treated using oral devices [43]. Oral devices have better portability and are not dependent on electricity. Oral devices are non-invasive and stabilize the mandible and the pharyngeal structures during sleep [44]. One of the most commonly used oral devices is the mandibular advancement device (MAD) [40]. This device has been manufactured with many different variations, including titratable or not-titratable. If the device is titratable, this is achieved by protrusion of the mandible [12]. Manufacture of the MAD is followed by a gradual titration of the device during the first months of use in order to achieve a therapeutic effect on the upper airway, while ensuring optimal compliance. A titration protocol has been evaluated for duo-block MADs but this titration protocol is only an example, as there is no standard titration protocol for the use of MADs in routine clinical practice. The level of protrusion was adjusted during each visit, with protrusion ranging from 45, 60, 75, to 90% of the maximum protrusive capability. The level of protrusion was set at 60% as the baseline and was increased if a subjective improvement was noted by the patient. If the patient was uncomfortable or if side effects were observed, the level of protrusion was decreased [45]. This protrusion produces an increase in the volume of the upper airway lumen and helps prevent collapse [18, 23, 24, 29]. Oral devices can be customized or non-customized and titratable or non-titratable. The American Academy of Dental Sleep Medicine recommends customized, titratable oral devices for treatment of OSA [40]. However, dental side effects have been observed with MAD treatment, including lower incisor retroclination and upper incisor proclination. Variation in incisor inclination has a linear relationship with the level of protrusion included in the device during its manufacture. These changes in the inclination of the incisors are caused by the force of the lips, whereas the tongue showed minimal effects [46]. In addition, mesial movement of mandibular molars and canine inclusion has been reported when the MAD covers all the teeth, but this does not seem to decrease levels of adherence to treatment with the device. MADs can also produce changes in occlusal contacts and lead to the appearance of interproximal gaps. Furthermore, a decrease of 86% in overjet and overbite has been reported following MAD use. The use of morning occlusal guides has been recommended in order to prevent these side effects. Other potential side effects include irritation of the gingiva and tongue, but these effects are usually minor and can normally be solved with orthodontic wax or by adjusting the device. Changes have also been observed in amount of salivation, as well as an increased gag reflex and anxiety caused by the presence of the appliance in the mouth [46]. The level of mandibular protrusion is also important in preventing side effects, which can worsen with greater levels of protrusion [12]. Computerized tomography has found statistically significant changes in the volume of the upper airway with 50% and 70% of protrusion of the mandible and 70% protrusion has resulted in greater transversal dimension of the upper airway when compared with 50% protrusion. Further study of MAD designs is required to improve manufacture and to strike a balance between effectiveness and prevention of adverse effects [47].

A major limitation of the present systematic review with meta-analysis is that only published trials were found. This reflects the predisposition of the sciences to publish positive results and not to include non-beneficial ones. Further randomized trials are needed to verify the results, including studies with longer treatment times.

## Conclusion

Unlike diastolic pressure, only systolic pressure appears to be affected by the use of MADs, with this decrease being more pronounced at night and during longer treatment times.

## Data Availability

Data available upon request in line with relevant restrictions, e.g. privacy or ethical.
